# Study of the Utility and Impact of Plasma *p*‐tau181 Alzheimer's Biomarkers ‐ SUIT‐ABLE: Clinical Care Outcomes

**DOI:** 10.1002/alz70856_101304

**Published:** 2025-12-25

**Authors:** Megan Zhang, Abigail Perelman, Cameron Coykendall, Kristin Harkins, Jason Karlawish, Kyra O’Brien

**Affiliations:** ^1^ University of Pennsylvania, Philadelphia, PA, USA; ^2^ University of Pennsylvania Perelman School of Medicine, Philadelphia, PA, USA

## Abstract

**Background:**

Early and accurate diagnosis of Alzheimer's disease (AD) affords access to appropriate care. AD diagnostic biomarkers can facilitate early diagnosis. Blood‐based, or plasma, AD biomarkers are rapidly emerging and can enable wider access to AD biomarker testing and therefore early and accurate diagnosis. Plasma *p*‐tau181 testing is a promising blood‐based biomarker for Alzheimer's Disease (AD), offering a less invasive and more accessible alternative to imaging or spinal fluid biomarkers. While its sensitivity for amyloid pathology is well‐established, its impact on clinician decision‐making, diagnostic confidence, and care management remains underexplored. This study evaluates how plasma *p*‐tau181 results influence diagnosis, clinician diagnostic confidence, and other aspects of clinical decision‐making in a memory center.

**Method:**

The LucentAD plasma *p*‐tau181 test (Quanterix) was utilized with a cut‐off providing sensitivity of 97.3% and specificity of 52.8% for Alzheimer's disease (AD) diagnosis. Eligible patients had a syndromic diagnosis of mild cognitive impairment (MCI) or dementia, no prior biomarker‐based AD diagnosis, and enrolled with a care partner. Clinicians completed surveys pre‐ and post‐ plasma *p*‐tau181 testing. The co‐primary outcomes were change in diagnostic confidence, diagnosis, and patient management. Chart abstraction was conducted three months post‐disclosure.

**Result:**

Ninety‐nine participants were enrolled between September 2023 and November 2024. Of the 94 participants with plasma *p*‐tau181 results, 81.9% (*n* = 77) tested positive, and 18.1% (*n* = 17) tested negative. In the elevated plasma *p*‐tau181 result group, 8 participants’ diagnoses were reclassified from other diagnoses to AD and 4 changed from AD to other diagnoses. In the low plasma *p*‐tau181 result group, 4 participants moved from AD to non‐AD diagnoses. Clinician‐reported diagnostic confidence significantly increased following result disclosure (*p* < 0.001). Among 79 participants with completed chart reviews, 15.2% had medication adjustments, 7.6% had new consultations ordered, and 54.4% had additional diagnostic testing ordered after plasma *p*‐tau181 testing. Of those with an elevated plasma *p*‐tau181 result, 24.6% had a lumbar puncture for spinal fluid AD biomarker testing, and 13.1% had an amyloid PET scan. No patients with a low plasma *p*‐tau181 result underwent additional AD biomarker testing.

**Conclusion:**

Plasma *p*‐tau181 testing enhances clinician diagnostic confidence and informs diagnostic decisions, particularly in distinguishing between AD and non‐AD diagnoses.

